# Comparison of Dietary Quality Assessment Using Food Frequency Questionnaire and 24-hour-recalls in Older Men and Women

**DOI:** 10.3934/publichealth.2017.4.326

**Published:** 2017-07-03

**Authors:** Elizabeth Procter-Gray, Barbara Olendzki, Kevin Kane, Linda Churchill, Rashelle B. Hayes, Annabella Aguirre, Hyung-joo Kang, Wenjun Li

**Affiliations:** Health Statistics and Geography Lab, Division of Preventive and Behavioral Medicine, Department of Medicine, University of Massachusetts Medical School, Worcester, MA 01655, USA

**Keywords:** gender, FFQ, 24-hour dietary recalls, diet quality, misclassification, healthy eating

## Abstract

**Objectives:**

To examine the agreement in nutrient intake and alternative healthy eating indices (AHEI) between a self-administered Food Frequency Questionnaire (FFQ) and 24-hour recall (24HR) measurements of diet by gender, among older adults.

**Material and methods:**

This is a cross-sectional observational study of 105 men and 99 women aged 65 and older living in urban and rural neighborhoods in Worcester County, Massachusetts, USA. Participants were queried on diet using both FFQ and 24HR. The healthy eating classification was compared between the two instruments by gender.

**Results:**

For men, the mean ± SD of AHEI total score was 48.2 ± 12.3 based on FFQ versus 34.7 ± 10.2 based on 24HR. For women, the mean ± SD was 47.9 ± 10.1 based on FFQ versus 36.1 ± 10.0 based on 24HR. Using 32 as the cutoff (40% of maximum AHEI score), 9% of men and 7% of women were classified as eating unhealthy based on the FFQ, versus 47% of men and 38% of women based on 24HR. Compared to women, men had larger 24HR to FFQ discrepancies in the nuts and vegetable protein subscore and white/red meat ratio, and smaller discrepancy in alcohol beverages subscore.

**Conclusion:**

Agreements between FFQ and 24HR-based measures of diet quality were roughly comparable between men and women, though slightly better for women than men. Compared to 24HR, the FFQ tended to underestimate the proportions of older men and women classified as eating unhealthy and misclassified more men than women. Such limitations should be considered when the FFQ is used to study healthy eating in older age.

## 1. Introduction

Healthy eating is critical to the prevention and care of chronic diseases and disabilities in older age [1],[2]. Accurate and cost-effective assessment of dietary intake and diet quality may inform personalized nutrition counseling and help monitor trends in adherence to dietary guidelines.

Commonly used methods include the interviewer-administered 24-hour dietary recall interviews (24HR) and self-administered food frequency questionnaire (FFQ). The 24HR requires a professionally trained interviewer to administer multiple telephone interviews with the respondents to capture the intra-person variability of diet [3]. The interviewer-administered 24HR is often preferred as the “gold standard”, against which a FFQ is calibrated [4],[5]. The FFQ is a low cost alternative and a frequent measurement of choice for large scale epidemiologic and health promotion studies. A typical FFQ includes a finite list of foods and food groups from which participants quantitatively report how often each item was consumed over a specified period of time. Portion size is collected according to standardized portion sizes.

Dietary behaviors are complex, and dietary assessment is prone to errors [6]. The accuracy of dietary assessment may be influenced by a number of respondents' personal characteristics, such as gender, age, race, culture, education, income, language, home environment and living arrangements, cognitive function and memory [7], and nutrition literacy [8]. In addition, social desirability or pressure regarding body image and weight can also impede reporting accuracy, disproportionally affecting overweight women [9]. Because biology, behaviors and social desirability differ between men and women, the accuracy of instruments to measure their dietary intake could well differ. Therefore, it is important to investigate discrepancies in dietary quality assessment using different instruments and among different populations. For example, in-depth analysis is needed to better understand whether use of different dietary assessment tools would affect the validity of the analysis of gender differences in diet quality.

As a part of a larger study of health behaviors among older adults, we assessed the utility of the self-administered General Nutrition Assessment (GNA) FFQ of the Fred Hutchinson Cancer Research Center (FHCRC) [10] in community-based studies on healthy eating. Our previous analysis showed that the FFQ has limited ability to accurately assess nutrient intake among older Black women, and tends to underestimate racial differences in diet and healthy eating among urban older women when compared to 24HR [11]. In the current analysis, we examined the agreement in nutrient intakes and the alternative healthy eating indices (AHEI) [12] between the FFQ and 24HR measurements of diet among older men and women living in rural and urban neighborhoods.

## 2. Materials and Methods

### 2.1. Study settings and participant recruitment

The study protocol was approved by the University of Massachusetts Medical School Institute Review Board (Docket #: H-14793). From 2012 to 2014, we recruited community-dwelling older men (n = 111) and women (n = 103) living in Worcester County, Massachusetts, USA. Participants were recruited through presentations at community organization meetings, distribution of flyers, and direct mail. Promotional materials, interest surveys and brief presentations were offered at each venue and tailored to meet site preferences. Presentations were made at senior centers, veterans' organizations, and men's breakfasts at retirement villages to promote and give a brief description of the study. The opportunity to participate was offered, asking those who were interested to complete a form for contact. In addition, flyers were distributed at the public housing authority, fitness clubs and at health fairs. Once a prospective participant expressed interest, the program director or designee contacted the participant, explained the study, determined eligibility, and mailed the prospective participant a consent form.

To be eligible for the study, participants had to be age 65 or older, able to consent, English speaking, ambulatory with or without assistive devices, willing and able to perform all study-related activities independently or with a designated caregiver, and to pass cognitive function screening on the Short Portable Mental Status Questionnaire (SPMSQ) [13] with 3 or fewer errors.

Once consented, a baseline visit was scheduled for each participant. Multiple methods of participation were offered to participants, including a one-on-one site visit with a study team member at UMass Medical School, at a Senior Center, or at their home, according to participant preferences and needs.

Surveys completed at home were returned by mail using a study-provided pre-paid envelope. Each participant completed two batteries of survey instruments, which took about 2–3 hours per battery. The first battery included surveys of sociodemographics, health and health care, lifestyle factors, anxiety, lower extremity problems, and fall history and efficacy. In the week following the completion of the first battery, participants wore an accelerometer and Global Positioning System (GPS) device for 7 consecutive days and completed three 24-hour recalls of dietary intake within the 7 days. Subsequently, the second battery included written instruments on diet (FFQ), physical activity, food habits and food purchasing, activity time and place, neighborhood perceptions, and depression.

### 2.2. Survey of personal characteristics

**Table 1.** Characteristics of participants (Mean ± SD or percent).

*Characteristic*	*Overall (N = 204)*	*Men (N = 105)*	*Women (N = 99)*	*P-value for diff.^1^*
*Sociodemographic*				
Age (years)	73.8 ± 5.9	74.6 ± 6.4	73.0 ± 5.2	0.10
Range (minimum – maximum)	65–93	65–93	65–85	
Race (%White)	88.2	87.6	88.8	0.80
Education (years)	14.9 ± 2.8	14.9 ± 3.1	14.8 ± 2.3	0.84
Annual income < $50,000	42.1	35.0	49.5	**0.04**
Married or living with partner	68.1	80.0	55.6	**<0.001**
Living alone	24.5	15.2	34.3	**0.002**
*Health and healthcare*				
CESD Depression Scale	5.7 ± 6.1	5.2 ± 5.6	6.2 ± 6.6	0.43
Beck Anxiety Scale	4.4 ± 5.5	3.4 ± 5.0	5.5 ± 5.8	**<0.001**
Number of comorbid conditions^2^	1.9 ± 1.5	1.8 ± 1.4	2.1 ± 1.6	0.16
Currently taking ≥2 medications^3^	22.8	24.8	20.7	0.49
Heart or circulatory condition	46.1	46.7	45.5	0.86
Diabetes	12.3	17.1	7.1	**0.03**
Respiratory disease	12.3	7.6	17.2	**0.04**
Cancer	22.1	21.0	23.2	0.70
Rheumatoid arthritis	5.4	5.7	5.1	0.83
Osteoarthritis	14.2	10.5	18.2	0.12
Osteoporosis	11.8	3.8	20.2	**<0.001**
Poor vision	2.0	2.9	1.0	0.34
Physical limitations (ADL)	1.0	1.0	1.0	0.97
Hospitalized in past 3 mos.	5.9	4.8	7.1	0.48
Number of indoor falls in past 6 mos.	0.2 ± 0.7	0.2 ± 0.4	0.2 ± 1.0	0.81
Number of outdoor falls in past 6 mos.	0.2 ± 1.0	0.3 ± 1.3	0.2 ± 0.5	0.48
Tinetti Falls Efficacy Scale	11.7 ± 7.7	12.0 ± 9.5	11.3 ± 5.0	0.92
*Lifestyle*				
BMI (kg/m^2^)	26.8 ± 4.5	27.5 ± 4.3	26.1 ± 4.6	**0.009**
Current smoker	2.5	1.0	4.1	0.16
Weekly frequency of alcohol	2.0 ± 2.4	2.3 ± 2.5	1.7 ± 2.2	0.07
Car ownership by household	99.5	100	98.9	0.29
Paid or volunteer employment	24.1	24.8	23.5	0.83
Physical activity (1,000 daily steps)	4.08 ± 1.94	4.50 ± 2.08	3.68 ± 1.71	**0.003**
Weekly frequency of exercise activities	19.3 ± 12.2	21.6 ± 14.0	16.9 ± 9.4	**0.03**

^1^ Chi2 test for categorical and Wilcoxon rank sum test for continuous variables.

^2^ Number of comorbidities from the following: heart or circulatory conditions (stroke, ischemic attack, high blood pressure); respiratory (asthma, COPD); cancer or malignant tumor; rheumatoid arthritis (not including rheumatism); intestine or colon polyps or adenomas; gallbladder disease or gallstones; systemic lupus erythematosus; kidney or bladder stones; diabetes; cataracts; glaucoma; osteoporosis; and osteoarthritis.

^3^ Medications taken for any of the comorbid conditions listed above.

Data from the first battery of instruments is itemized in Table 1. Most characteristics were assessed by self-report using questionnaires designed by this study, along with a number of standardized instruments including the Tinetti Falls Efficacy Scale for fear of falling [14], Beck Anxiety Inventory [15], CES-D Depression Scale [16], Activities of Daily Living (ADL) for physical limitations, and the CHAMPS (Community Healthy Activities Model Program for Seniors) survey of frequency of exercise activities, both recreational and functional [17],[18]. Weight and height of each participant was obtained by self-report. Physical activity was measured objectively by an accelerometer (ActiGraph GT3X-Plus) worn by each participant during all waking hours for 7 consecutive days in the week following the completion of the first battery of questionnaires. A daily mean number of steps was calculated for each person, excluding any non-wearing days (i.e., if <10 steps were recorded).

### 2.3. Measurements of dietary intake

The participants were queried about their diet using the 24HR and then the GNA FFQ, all performed within 3 weeks of each other. Each participant received three unannounced computer-assisted 24HR (University of Minnesota Nutrition Coordinating Center version NDSR 2011), conducted on randomly selected days within a 1-week period (two weekdays and one weekend) [19]–[21]. The GNA FFQ was developed by the NASR of the FHCRC, and is described elsewhere [11]. In summary, the GNA FFQ is originally based on the WHI FFQ, using the same format and analysis algorithms, and was updated in late 2010. The GNA FFQ relies upon the University of Minnesota Nutrition Data Systems for Research (NDSR) software version 2014 for data entry and nutrient analysis [20],[22].

Dietary outcomes of primary analytic interest in our study included average daily total caloric intake and measures of dietary quality, including consumption of fruits and vegetables, legumes, nuts, averaged daily intake of total protein, fats (saturated, poly- and monounsaturated fats, trans-fats), types of carbohydrates, fibers, and micronutrients (such as sodium and calcium).

We calculated healthy eating scores to compare the ability of the 24HR and FFQ instruments to evaluate dietary quality. First, an alternate healthy eating index (24HR AHEI) was calculated for each participant based on their 24HR intake, modifying the formula developed by the USDA Center for Nutrition Policy and Promotion [23]. As in prior studies, we did not include multivitamin intake in total score due to our interest in diet quality associated with food eating behaviors [24],[25]. The overall index had a total possible score ranging from 0 to 80 points, with higher scores indicating better overall dietary quality as it relates to morbidity and mortality of chronic diseases. Dietary component subscores were calculated for 8 components, including vegetables, fruits, nuts and legumes, ratio of white to red meat, cereal fiber, alcohol, trans fats, and ratio of polyunsaturated to saturated fats. Each component score had a scoring range of 0 to 10 points. In parallel, a FFQ-based AHEI score was calculated for each participant using the same 8 component scores. Comparable measures were available in the 24HR and FFQ data for all components except for the nuts and legumes subscore. Servings per day of nuts and legumes were not directly available from the FFQ, so this component score was estimated from the vegetable protein intake reported in the FFQ and scaled to match the point range of the same component in the 24HR AHEI. Total AHEI scores were categorized as “poor” in nutritional value if less than 40% of the maximum score, i.e., <32 points, following the procedure of Rehm and associates [26].

### 2.4. Statistical analysis

Gender differences in sociodemographic, physical and mental health, and lifestyle factors were evaluated using Chi-squared tests for percentages or Wilcoxon rank-sum tests for continuous variables (because many of the variables have skewed distributions). We also divided the participants into quartiles based on the absolute value of discrepancy between their total 24HR AHEI score and their total FFQ AHEI score. We then compared the characteristics of individuals in the quartile with greatest discrepancies with those in the lowest-discrepancy quartile in a supplementary table.

Dietary intake values of 48 nutrient items were compared by gender within both the FFQ and 24HR measurements using unadjusted linear regression models. For each nutrient item, the Pearson's product moment correlation (rho) between individuals' FFQ and 24HR measurements was estimated by gender, along with its 95% confidence interval based on Fisher's transformation. Rhos for each nutrient item were tested for equality between genders. Actual discrepancies between the FFQ and 24HR measurements were then examined item by item. For each nutrient item and each participant, the raw discrepancy was calculated (24HR measure − FFQ measure) as well as percentage discrepancy [absolute value (100 × (24HR − FFQ) / 24HR))]. The mean percentage discrepancies were compared by gender using linear regression models (1) unadjusted; and (2) adjusted for age, income, and education.

AHEI total scores and component subscores were summarized and compared for male to female differences using the Wilcoxon rank-sum test. Rhos between the FFQ- and 24HR-based scores were also estimated by gender. The mean differences between the 24HR- and FFQ-based AHEI scores were calculated. These were compared for gender differences using unadjusted linear regression models. Kappa scores and percent agreement were calculated for the classification of total AHEI scores into poor *vs.* better dietary quality by the FFQ *vs.* 24HR method. A scatter plot along with fitted linear regression lines and AHEI cutoff points at 32 was drawn to illustrate the gender differences in the distributional characteristics, correlations between FFQ- and 24HR-based AHEI scores, and misclassification of poor *vs.* better diet quality.

## 3. Results

### 3.1. Participant characteristics

Participants who completed both dietary assessments included 105 men and 99 women. They had a mean (SD) age of 73.8 (5.9), had 14.9 (2.8) years of education, and 88.2% were White (Table 1). Nearly all (97.5%) were current non-smokers and owned a car (99.5%). Compared to the men, higher proportions of the women had an annual household income below $50,000 and were living alone and/or unmarried. Women had, on average, lower levels of physical activity as measured by step counts and self-reported exercise frequencies. Women also had slightly but significantly lower BMI, a lower frequency of drinking alcoholic beverages, and higher levels of anxiety. Larger percentages of women than men had osteoporosis and respiratory disease, while more men had diabetes.

### 3.2. Dietary intake

Men and women differed significantly in their intake of many nutrients, according to both the FFQ and 24HR, with men consuming more daily calories, and thus having higher intakes of carbohydrates, proteins, and fats, and many micronutrients (Table 2). Men also consumed more caffeine and approximately twice as much alcohol as women, but had somewhat lower levels of vitamin A/beta carotene, lutein/zeaxanthin, and vitamin K in their diets. The 24HR appeared to be more sensitive to the gender differences than the FFQ. For instance, the 24HR estimated that men consumed 479 more calories than women *vs.* 287 more according to the FFQ. Similarly, the FFQ slightly underestimated differences in most macro- and micronutrient intakes relative to the 24HR, although there was good agreement between the two measures on the direction of the gender differences.

Examination of the correlations (rho) between FFQ and 24HR measurements revealed modest gender differences in a number of energy and nutrient intake measures (Table 3). The mean difference between male and female correlation coefficients for FFQ-24HR agreement was 0.07, with men having a mean correlation of 0.34 and women 0.42. Men had a “strong correlation” of 0.5 or greater for 4 of the 48 pairs, while women had strong correlations for 14 pairs. Statistically significant male to female differences were found in the correlations in reported percentage of calories from total fat, saturated fat, monounsaturated fat, and trans-fats; several vitamins (B6, folate, and E); and calcium. Notably, we found no statistically significant correlations between the FFQ and 24HR measurements of folate, vitamin B6, and vitamin E for men; percent of calories from trans-fats for women; and galactose (milk sugar) for both men and women.

Averaging, by gender, each individual's reporting discrepancy [24HR measure − FFQ measure] for each nutrient showed little male to female difference (Table 4). There were only three nutrients (of 48) for which one gender had a significantly (*p* < 0.05) greater percent discrepancy than the other in the crude analysis. In one of these cases (for percent of calories from protein), women had greater discrepancy between the two instruments, and for total folate and glycemic load, the men had a greater discrepancy. Adjusting for age, income, and education did not appear to have a large or univalent effect on the gender difference.

**Table 2.** FFQ- and 24-hour-recall-based daily energy and nutrient intakes by gender.

*Energy and nutrient intake*	*FFQ*					*24-HR*				
	*Men*		*Women*		*P for diff.*	*Men*		*Women*		*P for diff.*
	*Mean*	*SD*	*Mean*	*SD*		*Mean*	*SD*	*Mean*	*SD*	
*Primary energy sources*										
Total energy (kcal)	1782.1	666.7	1495.1	574.8	**0.001**	2049.3	568.2	1570.1	417.8	**<0.001**
Total fat (g)	61.1	26.8	52.5	26.8	**0.02**	77.4	29.5	58.9	21.3	**<0.001**
Total carbohydrate (g)	223.0	103.5	185.4	73.0	**0.003**	242.0	80.9	195.5	55.4	**<0.001**
Total protein (g)	71.6	28.9	64.6	29.7	0.09	82.8	28.6	63.8	18.4	**<0.001**
Animal protein (g)	47.6	23.6	43.1	24.4	0.18	54.1	27.6	40.9	17.2	**<0.001**
Vegetable protein (g)	23.9	10.9	21.6	9.6	0.11	28.6	9.9	22.9	8.5	**<0.001**
% Calories from fat	31.6	6.4	31.6	7.1	0.99	33.0	6.1	32.3	6.3	0.39
% Calories from carbohydrate	49.8	8.3	51.0	8.4	0.32	46.7	8.6	48.8	7.1	0.06
% Calories from protein	16.7	3.4	17.5	3.5	0.08	16.6	3.9	16.8	4.0	0.71
*Fats*										
% Calories from SFA	10.2	2.6	9.9	2.7	0.53	11.4	3.0	11.1	3.0	0.38
% Calories from MUFA	11.4	2.6	11.3	2.9	0.88	11.6	2.7	11.3	2.8	0.32
% Calories from PUFA	7.3	2.1	7.6	2.4	0.35	7.2	2.3	7.3	2.3	0.75
% Calories from TRANS	1.1	0.4	1.0	0.4	0.08	1.2	0.6	1.1	0.6	0.16
Omega-3 fatty acids (g)	1.5	0.7	1.3	0.6	**0.04**	1.7	0.9	1.7	0.9	0.57
PUFA:SFA ratio	0.8	0.2	0.8	0.3	0.27	0.7	0.3	0.8	0.4	0.16
Cholesterol (mg)	236.2	133.2	188.1	106.7	**0.005**	302.3	182.0	212.5	107.4	**<0.001**
*Carbohydrates*										
Total sugars (g)	112.6	66.6	92.5	43.4	**0.01**	102.1	48.1	84.1	34.5	**0.002**
Fructose (g)	26.2	23.0	19.2	11.4	**0.01**	19.5	13.0	15.7	8.6	**0.015**
Galactose (g)	0.4	0.3	0.5	0.3	0.24	0.2	0.4	0.4	1.0	0.16
Glucose (g)	26.3	21.5	19.3	11.2	**0.004**	20.6	13.3	16.1	8.3	**0.004**
Lactose (g)	18.5	16.1	16.6	14.2	0.38	13.0	9.9	11.0	8.6	0.13
Glycemic load	107.4	51.8	84.0	35.4	**<0.001**	130.7	48.7	104.4	31.8	**<0.001**
*Fiber*										
Total dietary fiber (g)	18.9	8.5	17.9	6.9	0.38	20.8	7.0	18.9	7.5	0.07
Soluble dietary fiber (g)	6.8	3.3	5.7	2.3	**0.005**	7.7	2.5	6.3	2.4	**<0.001**
Insoluble dietary fiber (g)	12.1	5.7	12.3	5.2	0.80	13.0	5.2	12.6	5.8	0.58
*Vitamins*										
Total Vitamin A activity (IU)	8393.6	5531.3	9591.7	6114.6	0.14	8326.3	5179.2	9020.7	6567.9	0.40
Thiamin (vitamin B1) (mg)	1.5	0.6	1.3	0.5	**0.002**	1.8	0.5	1.4	0.4	**<0.001**
Vitamin B-6 (mg)^1^	1.8	0.7	1.7	0.7	0.16	2.1	0.8	1.7	0.7	**0.001**
Vitamin B-12 (cobalamin) (mcg)	5.7	4.1	4.8	3.4	0.11	5.4	2.9	3.8	2.1	**<0.001**
Total folate (mcg)	399.9	193.6	346.5	153.4	**0.03**	427.3	172.6	357.5	131.6	**0.001**
Dietary folate equivalents (mcg)	499.5	236.7	430.7	200.4	**0.03**	573.4	280.6	460.8	193.3	**0.001**
Vitamin C (ascorbic acid) (mg)	126.0	133.9	108.1	86.8	0.26	85.5	53.2	91.6	62.5	0.45
Vitamin D (calciferol) (mcg)	6.2	4.4	5.5	3.8	0.23	5.8	4.0	4.9	4.0	0.08
Vitamin E (IU)	11.9	7.9	11.9	7.7	0.98	12.8	7.5	12.8	8.1	0.99
Vitamin K (phylloquinone) (mcg)	109.0	81.7	116.0	73.4	0.52	123.7	118.8	132.3	120.3	0.61
*Carotenoids*										
Beta-carotene (mcg)^2^	3554.2	2700.1	4422.4	3092.8	**0.03**	3484.8	2720.3	4178.5	3546.8	0.12
Lutein + Zeaxanthin (mcg)	2147.0	1960.4	2305.6	1741.7	0.54	1904.5	2299.9	2443.8	2665.6	0.12
Lycopene (mcg)	6178.4	4254.2	5885.3	5935.4	0.68	4448.5	4620.3	3753.9	4314.3	0.27
*Minerals*										
Calcium (mg)	980.5	581.4	877.5	469.7	0.17	930.7	318.9	743.0	257.1	**<0.001**
Iron (mg)	12.9	5.0	11.3	5.1	**0.03**	15.4	5.5	12.5	4.2	**<0.001**
Magnesium (mg)	295.2	121.8	277.0	105.8	0.26	311.5	89.8	267.8	92.8	**0.001**
Potassium (mg)	2929.3	1338.8	2641.6	1038.7	0.09	2875.5	803.5	2412.6	744.9	**<0.001**
Selenium (mcg)	105.2	41.5	93.7	41.1	**0.05**	119.6	42.1	89.5	27.3	**<0.001**
Sodium (mg)	2634.4	1055.0	2309.4	960.1	**0.02**	3000.7	950.6	2396.0	709.2	**<0.001**
*Other*										
Alcohol (g)	9.9	13.0	5.7	9.4	**0.009**	10.8	14.5	5.1	9.4	**0.002**
Caffeine (mg)	201.4	178.6	158.2	136.4	0.06	179.9	143.8	131.0	106.6	**0.006**
Water (g)	2421.0	1205.8	2252.3	838.4	0.25	2280.8	684.7	1979.6	575.9	**0.001**
Total grams	2778.0	1309.2	2554.9	899.3	0.16	2666.4	731.2	2284.7	592.5	**<0.001**

Sample sizes for both FFQ and 24HR measures in Tables 2–4: N = 105 men and 99 women except for calories and percent of calories for which N = 103 men and 97 women. IU = International Unit. TRANS = trans-fatty acids. MUFA = monounsaturated fatty acids. PUFA = polyunsaturated fatty acids. SFA = Saturated fatty acids. ^1^ Vitamin B-6 includes pyridoxine, pyridoxyl and pyridoxamine. ^2^ Beta-carotene including provitamin A carotenoid.

**Table 3.** Gender-specific Pearson correlation coefficients and associated 95% confidence intervals (CI) between FFQ- and 24HR-based measures of energy and nutrient intake.

*Energy and nutrient intake*	*Men*	*Women*	*Diff.*	*P for diff.*
*Primary energy sources*				
Total energy (kcal)	0.27 (0.08, 0.44)	0.39 (0.21, 0.55)	−0.12	0.35
Total fat (g)	0.35 (0.17, 0.51)	0.50 (0.33, 0.63)	−0.15	0.20
Total carbohydrate (g)	0.37 (0.19, 0.52)	0.32 (0.14, 0.49)	0.04	0.69
Total protein (g)	0.37 (0.19, 0.52)	0.46 (0.29, 0.60)	−0.10	0.44
Animal protein (g)	0.46 (0.29, 0.60)	0.52 (0.36, 0.65)	−0.06	0.58
Vegetable protein (g)	0.37 (0.19, 0.52)	0.41 (0.24, 0.57)	−0.05	0.74
% Calories from fat	0.41 (0.23, 0.56)	0.61 (0.46, 0.72)	−0.20	**0.05**
% Calories from carbohydrate	0.46 (0.29, 0.60)	0.55 (0.39, 0.67)	−0.09	0.40
% Calories from protein	0.57 (0.42, 0.69)	0.41 (0.23, 0.57)	0.15	0.14
*Fats*				
% Calories from SFA	0.37 (0.19, 0.53)	0.60 (0.45, 0.71)	−0.23	**0.03**
% Calories from MUFA	0.31 (0.12, 0.48)	0.57 (0.42, 0.69)	−0.26	**0.02**
% Calories from PUFA	0.47 (0.31, 0.61)	0.40 (0.21, 0.55)	0.08	0.55
% Calories from TRANS	0.41 (0.24, 0.56)	0.16 (−0.04, 0.34)	0.26	**0.05**
Omega-3 fatty acids (g)	0.29 (0.11, 0.46)	0.31 (0.12, 0.48)	−0.02	0.88
PUFA:SFA ratio	0.44 (0.27, 0.59)	0.50 (0.34, 0.64)	−0.06	0.59
Cholesterol (mg)	0.45 (0.29, 0.59)	0.42 (0.25, 0.57)	0.03	0.79
*Carbohydrates*				
Total sugars (g)	0.36 (0.18, 0.51)	0.33 (0.14, 0.49)	0.03	0.81
Fructose (g)	0.31 (0.13, 0.47)	0.31 (0.12, 0.48)	0.00	1.00
Galactose (g)	0.10 (−0.09, 0.29)	−0.02 (−0.22, 0.18)	0.12	0.40
Glucose (g)	0.28 (0.09, 0.45)	0.30 (0.11, 0.47)	−0.02	0.88
Lactose (g)	0.39 (0.22, 0.54)	0.59 (0.44, 0.70)	−0.19	0.06
Glycemic load	0.38 (0.20, 0.53)	0.32 (0.13, 0.49)	0.06	0.63
*Fiber*				
Total dietary fiber (g)	0.41 (0.24, 0.56)	0.34 (0.16, 0.51)	0.07	0.57
Soluble dietary fiber (g)	0.46 (0.30, 0.60)	0.32 (0.13, 0.48)	0.15	0.24
Insoluble dietary fiber (g)	0.43 (0.27, 0.58)	0.38 (0.20, 0.54)	0.05	0.67
*Vitamins*				
Total Vitamin A activity (IU)	0.30 (0.11, 0.46)	0.37 (0.18, 0.53)	−0.07	0.58
Thiamin (vitamin B1) (mg)	0.26 (0.07, 0.43)	0.33 (0.14, 0.49)	−0.07	0.59
Vitamin B-6 (mg)^1^	0.17 (−0.02, 0.35)	0.55 (0.40, 0.67)	−0.38	**<0.01**
Vitamin B-12 (cobalamin) (mcg)	0.23 (0.04, 0.40)	0.46 (0.29, 0.61)	−0.23	0.06
Total folate (mcg)	0.08 (−0.11, 0.27)	0.44 (0.27, 0.59)	−0.36	**<0.01**
Dietary folate equivalents (mcg)	0.08 (−0.11, 0.27)	0.46 (0.29, 0.60)	−0.38	**<0.01**
Vitamin C (ascorbic acid) (mg)	0.47 (0.30, 0.60)	0.57 (0.42, 0.69)	−0.11	0.33
Vitamin D (calciferol) (mcg)	0.53 (0.38, 0.66)	0.36 (0.17, 0.52)	0.17	0.13
Vitamin E (IU)	0.17 (−0.03, 0.35)	0.68 (0.56, 0.78)	−0.52	**<0.01**
Vitamin K (phylloquinone) (mcg)	0.26 (0.08, 0.43)	0.41 (0.23, 0.56)	−0.15	0.23
*Carotenoids*				
Beta-carotene (mcg)^2^	0.34 (0.16, 0.50)	0.35 (0.16, 0.51)	−0.01	0.94
Lutein + Zeaxanthin (mcg)	0.20 (0.01, 0.38)	0.43 (0.25, 0.58)	−0.22	0.07
Lycopene (mcg)	0.37 (0.19, 0.52)	0.26 (0.06, 0.43)	0.11	0.39
*Minerals*				
Calcium (mg)	0.34 (0.16, 0.50)	0.58 (0.44, 0.70)	−0.24	**0.03**
Iron (mg)	0.25 (0.07, 0.43)	0.36 (0.18, 0.52)	−0.11	0.39
Magnesium (mg)	0.33 (0.15, 0.49)	0.42 (0.25, 0.57)	−0.10	0.46
Potassium (mg)	0.34 (0.16, 0.50)	0.38 (0.19, 0.53)	−0.04	0.75
Selenium (mcg)	0.23 (0.04, 0.40)	0.44 (0.26, 0.59)	−0.21	0.09
Sodium (mg)	0.22 (0.03, 0.40)	0.37 (0.19, 0.53)	−0.15	0.25
*Other*				
Alcohol (g)	0.61 (0.47, 0.72)	0.67 (0.54, 0.76)	−0.06	0.47
Caffeine (mg)	0.61 (0.48, 0.72)	0.50 (0.34, 0.64)	0.11	0.26
Water (g)	0.33 (0.15, 0.49)	0.38 (0.19, 0.53)	−0.04	0.68
Total grams	0.33 (0.14, 0.49)	0.34 (0.15, 0.50)	−0.02	0.94
*Summary statistics of rho*				
Means	0.34	0.42	−0.07	
Medians	0.35	0.41	−0.06	
Minimum	0.08	−0.02	−-0.52	
Maximum	0.61	0.68	0.26	

IU = International Unit. TRANS = trans-fatty acids. MUFA = monounsaturated fatty acids. PUFA = polyunsaturated fatty acids. SFA = Saturated fatty acids. ^1^ Vitamin B-6 includes pyridoxine, pyridoxyl and pyridoxamine. ^2^ Beta-carotene including provitamin A carotenoid.

**Table 4.** Gender differences in 24HR-FFQ discrepancies in energy and nutrient intakes.

*Energy and nutrient intake*	*Raw discrepancies (mean±SD)^1^*		*Percent (%) discrepancies^2^*		*Male-female diff. in % disc.*	
	*Men*	*Women*	*Men*	*Women*	*Crude*	*Adj.^3^*
*Primary energy sources*						
Total energy (kcal)	267 ± 749	75 ± 563	31.5	27.3	4.2	3.6
Total fat (g)	16.3 ± 32.1	6.4 ± 24.6	37.0	33.8	3.2	3.3
Total carbohydrate (g)	19.0 ± 106	10.2 ± 76.0	36.3	30.3	6.0	5.4
Total protein (g)	11.2 ± 32.4	−0.8 ± 26.8	32.9	32.0	0.9	1.5
Animal protein (g)	6.5 ± 26.9	−2.2 ± 21.4	38.7	41.7	−3.0	−2.8
Vegetable protein (g)	4.7 ± 11.7	1.3 ± 9.8	37.0	34.7	2.3	2.6
% Calories from fat	1.5 ± 6.8	0.7 ± 6.0	16.7	15.3	1.4	2.6
% Calories from carbohydrate	−3.2 ± 8.8	2.2 ± 7.5	18.3	13.2	5.1	4.8
% Calories from protein	0.0 ± 3.4	−0.7 ± 4.1	16.3	20.6	−4.3 *	−3.5
*Fats*						
% Calories from SFA	1.3 ± 3.2	1.1 ± 2.5	22.8	20.3	2.5	3.8
% Calories from MUFA	0.3 ± 3.1	0.0 ± 2.7	21.2	20.1	1.1	2.1
% Calories from PUFA	−0.2 ± 2.2	−0.4 ± 2.6	28.5	29.9	−1.4	−2.1
% Calories from TRANS	0.1 ± 0.6	0.1 ± 0.7	47.4	49.4	−2.0	3.8
Omega-3 fatty acids (g)	0.3 ± 1.0	0.4 ± 1.0	49.5	46.0	3.5	1.4
PUFA:SFA ratio	−0.1 ± 0.3	0.0 ± 0.3	42.9	40.2	2.7	0.1
Cholesterol (mg)	66.1 ± 170	24.5 ± 115	47.3	43.3	4.0	5.6
*Carbohydrates*						
Total sugars (g)	−10.5 ± 66.9	−8.4 ± 45.8	49.5	48.1	1.4	0.3
Fructose (g)	−6.6 ± 22.6	−3.4 ± 12.0	107.6	81.6	26.0	28.8
Galactose (g)	−0.2 ± 0.5	−0.1 ± 1.1	487.0	506.2	−19.2	23.6
Glucose (g)	−5.7 ± 21.9	−3.2 ± 11.8	89.3	74.5	14.8	18.8
Lactose (g)	−5.6 ± 15.2	−5.7 ± 11.5	160.7	533.7	−373.0	−327.0
Glycemic load	23.4 ± 56.1	20.4 ± 39.2	38.7	30.7	8.0 *	7.5
*Fiber*						
Total dietary fiber (g)	1.9 ± 8.5	1.0 ± 8.3	35.3	34.6	0.7	0.2
Soluble dietary fiber (g)	0.9 ± 3.1	0.6 ± 2.8	34.6	31.9	2.7	2.5
Insoluble dietary fiber (g)	0.9 ± 5.8	0.3 ± 6.2	39.1	41.3	−2.2	−3.9
*Vitamins*						
Total Vitamin A activity (IU)	−67 ± 6354	−571 ± 7150	69.5	75.0	−5.5	−9.4
Thiamin (vitamin B1) (mg)	0.2 ± 0.7	0.1 ± 0.6	34.2	31.6	2.6	4.2
Vitamin B-6 (mg)^4^	0.2 ± 1.0	0.0 ± 0.7	37.6	35.5	2.1	1.7
Vitamin B-12 (cobalamin) (mcg)	−0.3 ± 4.5	−1.0 ± 3.1	56.9	90.1	−33.2	−50.2
Total folate (mcg)	27.5 ± 248	10.9 ± 152	44.5	34.1	10.4 *	9.1
Dietary folate equivalents (mcg)	73.9 ± 352	30.1 ± 204	45.6	35.7	9.9	8.8
Vitamin C (ascorbic acid) (mg)	−40.5 ± 119	−16.5 ± 72.3	87.1	83.5	3.6	2.7
Vitamin D (calciferol) (mcg)	−0.4 ± 4.1	0.7 ± 4.4	65.7	77.2	−11.5	−11.4
Vitamin E (IU)	0.9 ± 9.9	0.9 ± 6.3	56.7	41.7	15.0	25.2 *
Vitamin K (phylloquinone) (mcg)	14.7 ± 125	16.3 ± 113	78.9	88.4	−9.5	−14.6
*Carotenoids*						
Beta-carotene (mcg)^5^	−69.4 ± 3121	−244 ± 3807	144.2	142.4	1.8	−0.4
Lutein + Zeaxanthin (mcg)	−243 ± 2705	138 ± 2487	118.4	109.9	8.5	1.1
Lycopene (mcg)	1730 ± 5011	2131 ± 6379	527.7	1762.0	−1235	−1060
*Minerals*						
Calcium (mg)	−49.8 ± 559	−131 ± 382	43.6	41.8	1.8	3.3
Iron (mg)	2.5 ± 6.4	1.2 ± 5.3	34.4	31.2	3.2	4.7
Magnesium (mg)	16.3 ± 125.3	−8.6 ± 107	34.1	29.9	4.2	3.7
Potassium (mg)	−53.7 ± 1309	−225 ± 1025	34.8	35.0	−0.2	0.3
Selenium (mcg)	14.4 ± 52.0	−4.1 ± 38.1	36.2	35.1	1.1	2.6
Sodium (mg)	366 ± 1253	93 ± 959	35.7	33.6	2.1	2.7
*Other*						
Alcohol (g)	0.9 ± 12.2	−0.4 ± 7.7	2741	1502	1238	1142
Caffeine (mg)	−21.5 ± 145	−27.7 ± 124	298.6	492.1	−194.0	−235.0
Water (g)	−140 ± 1172	−266 ± 820	39.2	37.7	1.5	0.3
Total grams	−112 ± 1275	−263 ± 893	36.0	34.8	1.2	0.5

IU = International Unit. TRANS = trans-fatty acids. MUFA = monounsaturated fatty acids. PUFA = polyunsaturated fatty acids. SFA = Saturated fatty acids.* *p* < 0.05, ** *p* < 0.01 as estimated by linear regression models. ^1^ Raw discrepancy = 24HR - FFQ. ^2^ Percent discrepancy = absolute value of 100 × (24HR − FFQ) / 24HR). ^3^ Adjusted for age, income and education. ^4^ Vitamin B-6 includes pyridoxine, pyridoxyl and pyridoxamine. ^5^ Beta-carotene including provitamin A carotenoid.

**Table 5.** Gender differences in Alternate Healthy Eating Index (AHEI) scores according to FFQ and 24HR (Mean ± SD).

*AHEI Score*	*FFQ*			*24HR*			*24HR-FFQ Discrepancy*		
	*Men*	*Women*	*P for diff.^1^*	*Men*	*Women*	*P for diff.^1^*	*Men*	*Women*	*P for diff.^2^*
*Average scores*									
Total score (80-point max.)	48.2 ± 12.3	47.9 ± 10.1	0.93	34.7 ± 10.2	36.1 ± 10	0.29	−13.5	−11.9	0.32
Range of total score	18.5–74.9	25.1–70.2		15.8–61.9	11.4–61.3				
Subscores (10-point max.)									
Vegetables	4.5 ± 2.6	4.7 ± 2.6	0.49	4.1 ± 2.2	4.2 ± 2.4	0.51	−0.4	−0.5	0.88
Fruit	5.2 ± 3.2	4.9 ± 2.5	0.70	4.2 ± 2.8	4.2 ± 2.5	0.85	−1.0	−0.7	0.35
Nuts and vegetable protein	5.1 ± 4.0	4.0 ± 3.9	0.07	3.5 ± 3.4	3.7 ± 3.6	0.90	−1.6	−0.3	**0.05**
White/red meat ratio	8.5 ± 3.3	9.4 ± 2.3	**0.04**	3.8 ± 3.1	5.6 ± 3.1	**0.001**	−4.8	−3.8	0.07
Cereal fiber	6.1 ± 2.4	5.8 ± 2.1	0.73	2.6 ± 2.4	2.3 ± 2.2	0.46	−3.5	−3.6	0.91
Alcoholic beverages ^3^	3.4 ± 3.5	3.3 ± 4.0	0.67	2.8 ± 3.2	1.7 ± 2.7	**0.01**	−0.6	−1.6	**0.01**
Percent of calories trans fat	8.3 ± 1.1	8.6 ± 1.0	0.14	5.8 ± 2.2	6.1 ± 2.1	0.50	−0.3	−0.2	0.54
Polyunsaturated/sat fat ratio	7.1 ± 2.2	7.2 ± 2.1	0.72	8.0 ± 1.4	8.3 ± 1.7	**0.02**	−1.3	−1.1	0.62
*Healthy eating classification (%)^4^*									
Poor (<32; i.e.,40% of max score)	8.6	7.1		46.7	37.8				
Intermediate (32–63.9)	81.9	88.8		53.3	62.2				
Good (≥64; 80% of max score)	9.5	4.1		0	0				
% “misclassified” as “not poor”	38.1	30.7							

Sample size is N = 105 for men and N = 98 for women.

^1^ *P*-values for differences estimated by Wilcoxon rank-sum test.

^2^ *P*-values for gender difference by t-test.

^3^ Men are required to have a higher alcohol intake than women (1.5–2.5 *vs.* 0.5–1.5 servings per day) for maximum score.

^4^ Conventional classifications according to Rehm et al (2016).

### 3.3. Healthy eating index

Alternate Healthy Eating Indices (AHEI) calculated from the FFQ-reported diet *vs.* the 24HR-reported diet are shown in Table 5. Men and women did not differ significantly from each other in total AHEI by either measure. Women had a significantly higher subscore for the ratio of white/red meat consumed according to both measures. Only the 24HR found subscore differences for alcohol (men higher) and polyunsaturated/saturated fat ratio (women higher). The greatest discrepancies between FFQ and 24HR (3.5 points or greater) were found in the subscores for white/red meat ratio and for cereal fiber in both genders. Compared to women, men had larger FFQ to 24HR discrepancies in the subscores for nuts and vegetable protein and for white/red meat ratio, while women had a larger discrepancy in the alcoholic beverages subscore. With respect to total AHEI scores, personal characteristics of participants in the highest 24HR-FFQ-discrepancy quartile did not differ greatly from those in the least-discrepancy quartile (Supplementary Table 1). Those with the greatest discrepancy were more physically active (more steps per day) and were more likely to be married, and less likely to live alone.

**Table 6.** Gender-specific Pearson correlation coefficients (95% confidence intervals) between FFQ- and 24HR-based Alternative Healthy Eating Indices (AHEI).

*AHEI scores*	*Men (N = 105)*	*Women (N = 98)*	*Gender diff.*	*P for diff.*
*Total score*	0.46 (0.29, 0.60)	0.55 (0.39, 0.67)	−0.09	0.40
*Subscores*				
Vegetables	0.41 (0.24, 0.56)	0.34 (0.15, 0.50)	0.08	0.57
Fruit	0.59 (0.45, 0.70)	0.51 (0.35, 0.65)	0.08	0.42
Nuts and vegetable protein	0.19 (−0.01, 0.37)	0.34 (0.15, 0.50)	−0.15	0.26
White/red meat ratio	0.21 (0.02, 0.39)	0.18 (−0.02, 0.36)	0.04	0.83
Cereal fiber	0.21 (0.02, 0.38)	0.36 (0.18, 0.53)	−0.16	0.25
Alcoholic beverages	0.69 (0.57, 0.78)	0.64 (0.51, 0.74)	0.05	0.53
Percent of calories trans fat	0.42 (0.25, 0.57)	0.18 (−0.02, 0.37)	0.24	0.06
Polyunsaturated/sat fat ratio	0.43 (0.26, 0.57)	0.37 (0.18, 0.53)	0.06	0.62

**Figure 1.**
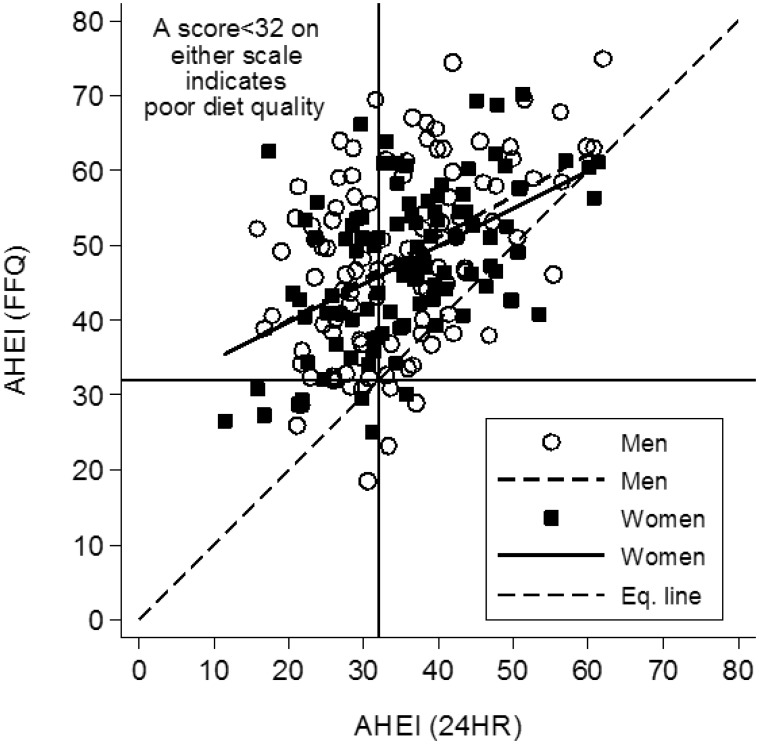
Relationship between FFQ- and 24HR-based Alternative Healthy Eating Index (AHEI) scores by gender. The light dashed line shows where points would fall if there were perfect agreement between the 24HR AHEI (horizontal axis) and the FFQ AHEI (vertical axis). Most points are above this line, indicating that the FFQ tends to award higher scores than the 24HR. The lines of best fit for men and women are also depicted and show the greatest departure from the equality line at the lowest AHEI scores, meaning that the FFQ overestimate of dietary quality relative to the 24HR is greatest among people with the poorest diets.

Women had a higher correlation between FFQ and 24HR-derived total AHEI scores, but men had higher correlations for six of eight subscores (Table 6), although none of these gender differences were statistically significant. For men and women combined the correlations were strongest for the subscores of alcoholic beverages, fruit, vegetables, and polyunsaturated to saturated fat ratio and weakest for the white to red meat ratio. The FFQ overestimated total AHEI relative to the 24HR by 13.5 points for men and 11.8 points for women (Table 5). Using 32 points as the cutoff (40% of maximum AHEI score), 47% of men and 38% of women were classified as having a poor diet based on 24HR versus 9% and 7% based on FFQ, respectively. The percent agreement between FFQ and 24HR AHEIs for poor diet classification, as determined by the kappa test, was 56% for men but 69% for women. As shown in Figure 1, while all FFQ AHEI scores tended to be higher than the corresponding 24HR AHEI scores (above the equality line), those in the lowest range tended to have the greatest discrepancy (i.e., line of best fit is farthest from the equality line in the lower region).

## 4. Discussion

A dietary assessment tool that is equally accurate and reliable for both men and women is critical to the studies of diet and eating behaviors in the general population. The GNA FFQ has been applied in numerous large scale studies [19],[27]–[32], primarily among post-menopausal women. This cost-efficient instrument has contributed new and important findings in older women's nutrition and health. It is of great interest to examine its utility in a broad variety of populations, such as studies on elder gender differences. Our analysis in a predominantly white, mixed rural-urban population in Central Massachusetts found that the FFQ was somewhat effective in identifying nutrient intake differences between the genders. However, the FFQ was less sensitive and detected a smaller magnitude of difference for total calories, total grams (volume of intake), many micronutrients, and most macronutrients as compared with the 24HR.

For the older women in our sample, the GNA FFQ and concurrent 24HR had an overall mean correlation of 0.42 for 48 nutrients. This was commensurate in accuracy with the original validation study results of the WHI FFQ against the 24HR. The correlation coefficients obtained by Kristal et al. [28]. for Whites (rho = 0.49) and by Patterson et al. [33] (0.45), are comparable to this. The men's mean rho of 0.34 raises the question of whether the FFQ could be a less accurate instrument for assessing men's diets in general, although this gender difference in rho was much less than some previously observed racial differences among older urban women by Olendzki et al. [11] (rho = 0.46 Whites and 0.23 Blacks) and Kristal et al. [28] (rho = 0.49 Whites and 0.26 Blacks). Our supplementary inter-quartile comparison of high-discrepancy *vs.* low-discrepancy participants found that living alone/unmarried was more common among those with the best agreement between their 24HR and FFQ total AHEI scores. Since this was also a more common characteristic among women than men in our study, it raises the question of whether living in a household where someone else may do the shopping and/or cooking, rather than gender itself, leads to greater disagreement between the two measures. Perhaps this factor should be considered when selecting a dietary evaluation instrument.

On an item-by-item basis both genders were similar in their 24HR-FFQ discrepancies. There were only three nutrients for which one gender had a significantly greater percent discrepancy than the other. This is very different from the results found by Olendzki et al [11]. For the same set of nutrients in a comparison between Black and White older women, there were 24 nutrients with significant White-Black differences in mean percent discrepancy, and in every case, Blacks had a greater discrepancy than Whites. Here again, as for the measurement correlations, the gender differences in FFQ-24HR agreement were much less than the previously observed racial differences.

For assessment of healthy eating status, the correlations between FFQ- and 24HR-based AHEI total scores were high for both genders (0.55 for women and 0.46 for men). However, the FFQ tended to overestimate AHEI scores and the proportion of participants designated as eating healthy for both men and women. Even though the men in our sample had only slightly lower mean AHEI scores than women according to the 24HR, the overestimation by the FFQ moved a larger proportion of men into the “intermediate” diet quality range from their “poor” status under the 24HR. As discussed by our previous analysis [11], this error may be especially serious in studies that include at-risk racial minority populations.

Although our analysis focused on the discrepancies in AHEI, similar analysis should be conducted to examine discrepancies in other well-known dietary quality indices such as HEI and DASH. Such analyses may inform users about the proper use of these instruments when studying disparities in diet and dietary behaviors. In addition to gender and racial differences, geographical and cultural differences may also need to be carefully examined to ensure the validity of diet quality assessment valid across geographic regions and cultural groups. We will address these issues in our future studies.

There are several strengths as well as weaknesses in the current study. The strengths include a relatively large, representative sample of community-dwelling men and women from diverse neighborhoods of low to high housing density. The timing of the two measurement surveys was less than three weeks apart for the majority of participants, limiting the impact of seasonal and typical dietary variation. The study also carefully measured a large number of sociodemographic (e.g., education and income), lifestyle and health characteristics of the participants, which allowed us to explore personal factors that may influence reporting accuracy and calculate covariate-adjusted gender differences. However, the present sample consisted of predominantly non-Hispanic white men and women, and the study findings cannot be generalized to racial and ethnic minorities. Both FFQ and 24HR are subject to recall accuracy and social desirability bias.

## 5. Conclusion

The GNA FFQ has played a vital role in discovery of nutrition-related risk factors for chronic diseases and injuries among older women. This analysis showed that agreements between FFQ- and 24HR-based measures of diet quality were roughly comparable for women and men. Therefore, its utility among older men could be considered in future large scale studies on both genders, at least for non-Hispanic white populations. Compared to 24HR, however, the FFQ tended to underestimate the proportions of older men and women classified as eating unhealthy, and a somewhat higher proportion of men were misclassified. The correlations for a number of energy and nutrient intake measures between FFQ and 24HR differ between men and women. Such limitations also should be considered when the FFQ is used to study healthy eating in older age.

**Supplementary Table 1:** Characteristics of participants (Mean ± SD or %), comparing those with the greatest *vs.* least FFQ-24HR discrepancy (absolute value) in total AHEI score.

*Characteristic*	*Overall study sample (N = 204)*	*Upper quartile of FFQ-24HR discrepancy (N = 51)*	*Lower quartile of FFQ-24HR discrepancy (N = 51)*	*p-value for difference ^1^*
*Sociodemographic*				
Age (years)	73.8 ± 5.9	73.4 ± 6.2	73.5 ± 5.7	0.86
Range (minimum – maximum)	65–93	65–89	65–91	
Race (%White)	88.2	88.2	86.0	0.74
Education (years)	14.9 ± 2.8	15.0 ± 2.8	14.3 ± 2.4	0.25
Annual income < $50,000	42.1	34.7	50.0	0.12
Married or living with partner	68.1	78.4	60.8	**0.05**
Living alone	24.5	13.7	33.3	**0.02**
*Health and healthcare*				
CESD Depression Scale	5.7 ± 6.1	5.9 ± 7.5	4.4 ± 4.2	0.93
Beck Anxiety Scale	4.4 ± 5.5	4.3 ± 4.7	4.6 ± 6.0	0.88
Number of comorbid conditions ^2^	1.9 ± 1.5	1.7 ± 1.4	2.1 ± 1.6	0.30
Currently taking ≥2 medications ^3^	22.8	26.0	22.0	0.64
Heart or circulatory condition	46.1	56.9	45.1	0.24
Diabetes	12.3	15.7	7.8	0.22
Respiratory disease	12.3	5.9	17.6	0.07
Cancer	22.1	15.7	23.5	0.32
Rheumatoid arthritis	5.4	3.9	5.9	0.65
Osteoarthritis	14.2	11.8	17.6	0.40
Osteoporosis	11.8	9.8	11.8	0.75
Poor vision	2.0	5.9	2.0	0.31
Physical limitations (ADL)	1.0	2.0	0.0	0.32
Hospitalized in past 3 mos.	5.9	2.0	5.9	0.31
Number of indoor falls in past 6 mos.	0.2 ± 0.7	0.2 ± 0.5	0.2 ± 0.4	0.97
Number of outdoor falls in past 6 mos.	0.2 ± 1.0	0.2 ± 0.4	0.1 ± 0.4	0.58
Tinetti Falls Efficacy Scale	11.7 ± 7.7	11.0 ± 3.2	11.9 ± 7.5	0.92
*Lifestyle*				
BMI (kg/m ^2^)	26.8 ± 4.5	27.4 ± 4.9	26.5 ± 3.6	0.71
Current smoker	2.5	4.0	2.0	0.57
Weekly frequency of alcohol	2.0 ± 2.4	2.3 ± 2.2	1.8 ± 2.3	0.11
Car ownership by household	99.5	100.0	97.8	0.30
Paid or volunteer employment	24.1	31.4	18.0	0.12
Physical activity (1,000 daily steps)	4.08 ± 1.94	4.58 ± 2.10	3.74 ± 2.14	**0.02**
Weekly frequency of exercise activities	19.3 ± 12.2	20.1 ± 12.2	18.2 ± 13.1	0.27

^1^ Chi2 test for categorical and Wilcoxon rank sum test for continuous variables. ^2^ Number of comorbidities from the following: heart or circulatory conditions (stroke, ischemic attack, high blood pressure); respiratory (asthma, COPD); cancer or malignant tumor; rheumatoid arthritis (not including rheumatism); intestine or colon polyps or adenomas; gallbladder disease or gallstones; systemic lupus erythematosus; kidney or bladder stones; diabetes; cataracts; glaucoma; osteoporosis; and osteoarthritis. ^3^ Medications taken for any of the comorbid conditions listed above.
